# Role of Self-Expandable Metal Stents in Acute Variceal Bleeding

**DOI:** 10.1155/2012/418369

**Published:** 2012-08-09

**Authors:** Fuad Maufa, Firas H. Al-Kawas

**Affiliations:** Division of Gastroenterology, Department of Medicine, MedStar Georgetown University Hospital, Georgetown University, 3800 Reservoir Road, NW, Washington, DC 20007, USA

## Abstract

Acute variceal bleeding continues to be associated with significant mortality. Current standard of care combines hemodynamic stabilization, antibiotic prophylaxis, pharmacological agents, and endoscopic treatment. Rescue therapies using balloon tamponade or transjugular intrahepatic portosystemic shunt are implemented when first-line therapy fails. Rescue therapies have many limitations and are contraindicated in some cases. Placement of fully covered self-expandable metallic stent is a promising therapeutic technique that can be used to control bleeding in cases of refractory esophageal bleeding as an alternative to balloon tamponade. These stents can be left in place for as long as two weeks, allowing for improvement in liver function and institution of a more definitive treatment.

## 1. Introduction

Acute variceal bleeding continues to be associated with significant mortality. Recently published randomized controlled trials have shown that mortality from acute variceal bleeding has decreased over the past two decades from 42% to 15%, but this figure is still remarkably high [1]. Current recommendations for the treatment of acute variceal bleeding are to combine hemodynamic stabilization, antibiotic prophylaxis, pharmacological agents, and endoscopic treatment [2]. Endoscopic therapy using band ligation combined with vasoactive drugs is highly effective. First line therapy however may fail to control bleeding or is associated with early rebleeding (within five days) in as many as 10–20% of patients [2]. These patients are treated with rescue therapies including balloon tamponade (BT), insertion of a transjugular intrahepatic portosystemic shunt (TIPS), or surgical shunts. 

## 2. Limitations of Current Rescue Therapies

### 2.1. Balloon Tamponade

Balloon tamponade (BT) using Sengstaken-Blakemore tube used to be the primary therapy prior to the availability of endoscopic methods. Sengstaken-Blakemore tube, was first described in 1950 [3], is a multi-luminal plastic tube with two inflatable balloons (esophageal and 250 cc gastric balloons). Minnesota tube is a modified Sengstaken-Blakemore tube with an esophageal suction port. Linton-Nachlas tube has a single 600 cc gastric balloon. BT is effective in controlling bleeding at least temporarily, in over 80% of patients [2, 4–7]. Bleeding recurs after deflation in over 50% of cases [2, 4]. The most common error limiting the efficacy of tamponade is failure to position the gastric balloon correctly at the gastroesophageal junction [8]. It is seldom necessary to inflate the esophageal balloon if the gastric balloon is correctly positioned. BT should be only used as temporary bridge to control massive bleeding until more definitive therapy could be instituted within 24 hours [7, 9]. Use of BT requires appropriate expertise. Rate of complications is increased and efficacy is limited if the tube is placed by inexperienced operator. BT is associated with fatal complications in 6–20% of cases [4]. The most serious complication is the esophageal rupture following inflation of gastric balloon in the esophagus [10, 11]. Proximal tube migration can lead to asphyxiation [12]. Prolonged inflation of esophageal and gastric balloon can lead to esophageal and gastric ulceration from pressure necrosis. Aspiration is frequent after tube insertion and occurs in 10–20% of patients. The presence and degree of encephalopathy may be a contributing factor. Aspiration may be prevented by elective endotracheal intubation prior to insertion of BT [6, 7]. The presence of the tube is highly unpleasant for the patient and repeated endoscopic examinations may be difficult. 

### 2.2. Surgical Management

Surgical management for bleeding esophageal varices falls into two categories: shunt and nonshunt procedures. Nonshunt procedures include esophageal transection and gastroesophageal devascularization. Shunt procedure is classified into selective shunts such as splenorenal shunts, partial shunts such as calibrated small-diameter portacaval shunts, and nonselective shunts such as portacaval shunts. Surgical procedures are used less frequently in the era of advanced endoscopic therapy, TIPS, and liver transplant. However, surgical intervention remains an important and effective treatment modality in selected patients [13]. Distal splenorenal shunts and nonselective shunts are very effective in controlling bleeding [4]. These procedures are more protective against rebleeding than gastroesophageal devascularization [13]. Modified Sugiura procedure (transabdominal gastroesophageal devascularization + esophageal stapled transection + splenectomy) can be a life-saving procedure in patients with anatomy unsuitable for shunt surgery or for patients treated in nonspecialized centers where surgical expertise for a shunt operation is not available [14]. Despite of effectiveness of surgical procedures, mortality remains high (45–75%) [4, 15] and hepatic encephalopathy continues to be an important complication even in selective shunt procedures [4]. The calibrated small-diameter portacaval shunt was reported to have lower rate of hepatic encephalopathy compared to nonselective shunt in one randomized clinical trial but this remains to be replicated by other investigators [16].

### 2.3. Transjugular Intrahepatic Portosystemic Shunt (TIPS)

TIPS is an intrahepatic shunt that is placed using a percutaneous approach. It connects the hepatic vein and intrahepatic branch of portal vein using an expandable metallic stent with a diameter of 8 to 12 mm. TIPS was introduced as an alternative to surgery in the 1990s and has replaced surgical shunts in most centers [17]. Current practice guidelines reserve TIPS for patients in whom hemorrhage from esophageal varices cannot be controlled or in whom bleeding recurs despite combined pharmacological and endoscopic therapy. TIPS is extremely effective in controlling variceal bleeding with a reported rate of immediate hemostasis of 95% and with rebleeding in only 18% of patients [18]. However, worsening of liver function and encephalopathy continue to be a problem and mortality remains high because of further deterioration due to liver failure [4]. In some studies, 30–35% of patients developed hepatic encephalopathy following TIPS [19]. The current use of TIPS as rescue therapy was challenged in a recent study where early use of TIPS in high-risk patients (Child-Pugh class C or those in class B who have persistent bleeding) was reported to be associated with improved outcome and reduction in mortality [20]. Insertion of TIPS is technically challenging and may not be available in some centers, thus necessitating the need to transfer a critically ill patient. Occlusion of the hepatic or portal veins may preclude TIPS placement. 

The previously listed limitations of the current rescue therapies have led to the continued search for other methods as rescue therapy for refractory esophageal variceal bleeding.

## 3. Self-Expandable Metal Stent in Treatment of**** Refractory Esophageal Variceal Bleeding

**Figure 1:**
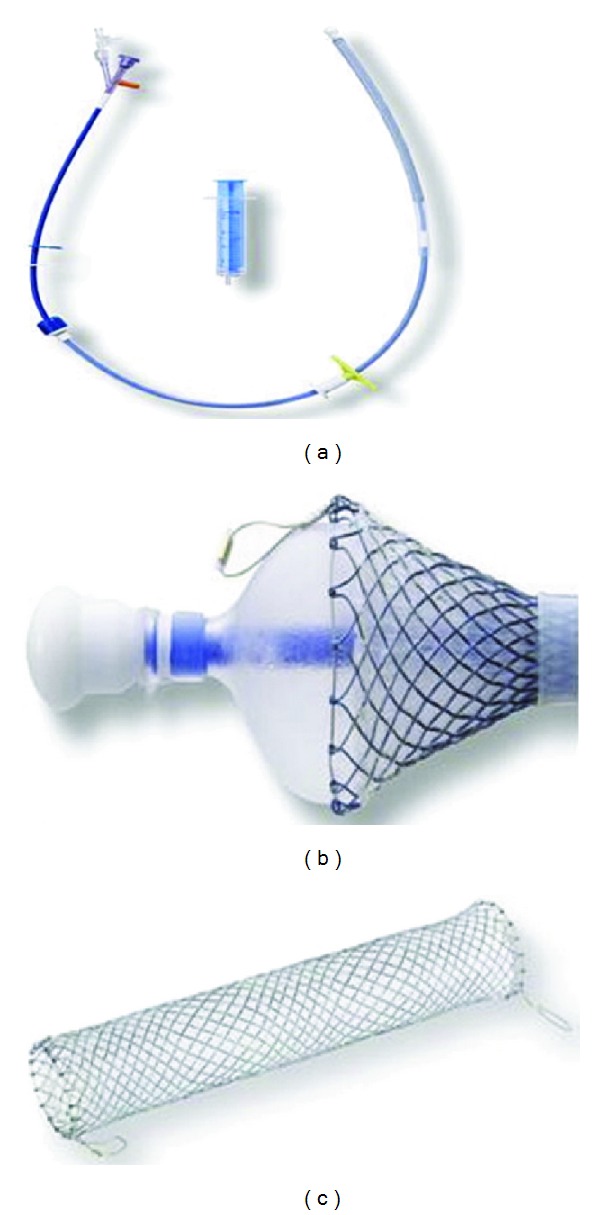
(a) The SX-Ella DANIS stent is supplied preloaded in an insertion device that has a 26 F diameter and is 60 cm long. (b) A balloon at the distal end of the insertion device (shown partially inflated) allows anchoring of the distal end of the stent at the cardia during deployment. (c) The fully deployed stent is 135 mm long and 25 mm wide. [Reprinted from [21]].

Self-expandable metal stents (SEMSs) are increasingly used in treatment of esophageal obstruction, stricture, leak, perforation, and tracheoesophageal fistula [22]. Anecdotal experience suggests that the covered version of these stents may be helpful in controlling bleeding esophageal varices. However, minimal published data is available and issues related to efficacy of insertion and safety need to be clarified. Recently, a specially designed SEMS for the treatment of refractory esophageal variceal bleeding became available [23]. The SX-Ella Danis stent (Ella-CS, Hradec Kralove, Czech Republic) is removable, covered SEMSs that can be deployed in the lower esophagus over an endoscopically placed guide wire without radiological assistance (Figure 1) [21]. The stent has atraumatic edges and radiological markers at both ends and at the midpoint to easily assess its position by a plain chest X-ray. Retrieval loops with gold markers at both stent ends allow the endoscopic extraction of the stent. In general, during endoscopy, a guide wire is placed in the stomach under direct vision and the endoscope is removed. The stent delivery device is then advanced over the guide wire into the stomach, and the distal portion of the stent delivery system is withdrawn to allow inflation of the gastric balloon. The gastric balloon is then inflated with air, and the whole delivery system is withdrawn until resistance is felt, which signifies that the balloon is impacting at the cardia. After stent deployment, the gastric balloon is deflated and the stent delivery system is withdrawn. The stent controls bleeding by tamponade of varices in the lower esophagus. The stent can be left in place for as long as two weeks. Stents can be removed using a special extractor device provided with the stent kit (PEX-Ella extractor device). Four published case series evaluated the effectiveness and safety of this stent in treatment of refractory esophageal variceal bleeding [21, 23–25]. “Refractory esophageal variceal bleeding” refers to ongoing bleeding despite pharmacological and endoscopy therapy. A summary of findings is listed in Table 1.

The initial pilot study by Hubmann et al. in 2006 reported the use of SEMS in 20 patients with massive ongoing esophageal variceal bleeding [23]. All patients failed prior endoscopic or pharmacological therapy. In this study, SEMSs were used as an alternative to balloon tamponade. Eight patients were Child-Pugh grade B and 12 grade C. Standard esophageal SEMS were used in the initial five patients. Choo stents (diameter 18 mm, length 140 mm; NES-18-080-070, M.I. Tech Co., Ltd) were used in two and the Ella-Boubela-Danis stent (diameter 20 mm, length 95 mm; Ella-CS, Hradec Kralove, Czech Republic) was used in three patients. In the remaining 15 patients, the newly designed Ella-Danis stent (diameter 25 mm, length 135 mm) was used. The stents were inserted using special introducer that allows placement of the stent without radiological or even endoscopic control. Correct insertion was accomplished by inflating the balloon at the distal end of the insertion device. The balloon was retracted to the cardia before release of the stent allowing correct positioning. After release of the stent, the balloon was deflated and the insertion device was removed. Upper endoscopy was performed after stent placement. A chest X-ray was obtained 12 hours after placement to confirm correct stent position. Stent placement was successful and bleeding was controlled immediately in all but one patient. This patient continued to bleed from gastric varices and underwent surgery (total gastrectomy and an open azygoportal disconnection) to control the bleeding. The remaining 19 patients were stable within two hours. No rebleeding occurred during 30-day followup. Stent migration to the stomach was reported in five patients with no apparent complications or rebleeding. Only 2/15 patients with the SX-Ella Danis stent had migration. Apparently, migrating stents were repositioned with endoscopy. All stents were extracted using standard endoscopy and a special foreign-body extractor (2–14 days after placement) with no complications. One patient was found to have a small ulceration in the distal esophagus. Two patients died 3 and 5 days after stent placement due to multiple organ failure. One of these patients had esophageal rupture caused by a Sengstaken tube used before the stent procedure. Neither of these patients had recurrent bleeding from the varices. After stent extraction, the remaining 18 patients underwent evaluation for definitive treatments. The main procedures in these patients were TIPS (5 patients), laparoscopic azygoportal disconnection (5 patients), band ligation (4 patients), and interventional radiography-guided coiling (1 patient). Liver transplant was eventually performed in three of these patients. 

Hubmann et al. published their extended series of 39 patients with massive ongoing esophageal variceal bleeding despite prior use of endoscopic or pharmacological therapy [24]. Results were similar to the previous report (20 of 39 patients were the same group of patients used in the previous report). In this study SEMS was again used as an alternative to balloon tamponade. SX-ELLA Danis stent was used. The technique of the implantation was similar to the previous published series. Stent placement was successful and uncomplicated for all patients. Bleeding was stopped in all patients. Stents were extracted with a special designed extractor. One patient was found to have a minor esophageal ulcer but no other local complications reported. Stent migration to the stomach was observed in seven patients. The 30-day mortality rate was 26.5%. None of the patients experienced bleeding recurrence. Definitive therapy was employed in most of the patients after stent extraction. The principal procedure was band ligation in 11 patients, TIPS insertion in 8 patients, and laparoscopic azygoportal disconnection in 5 patients. Two patients were put on a liver transplant list. 

Wright et al. reported their experience using the SX-Ella Danis stent in ten patients with variceal bleeding and contraindications to TIPS insertion or balloon tamponade [21]. The patients were not considered candidate for TIPS because of the multiple organ failure, severe liver disease, or the presence of hepatocellular carcinoma. Two patients had BT-induced esophageal tears. The stent was delivered using a technique similar to the previous studies. In one patient, the stent was placed without prior endoscopy because of severity of bleeding. Stents were placed successfully in 9 of the 10 patients. The failed deployment was caused by failure of the gastric balloon to inflate. Nine patients were actively bleeding at the time of stent insertion; in these patients, immediate control of bleeding was observed in 7 patients after stent insertion. In the remaining two patients, the source of bleeding was subsequently confirmed to be from gastric varices. Six of 9 successfully stented patients survived the acute bleeding episode. No information was given about stent migration. In one patient, the stent was removed under fluoroscopic control. In the remaining patients, stents were extracted successfully with the PEX-Ella extractor device at a median of 9 days (range 6–14 days). No major local complications were reported. One patient had esophageal ulcer related to the proximal end of the stent. Using Baveno IV consensus criteria, failure to control bleeding was observed in 3 patients (one patient died of multiple organ failure two days after stent insertion and two patients died of exsanguination). The 42-day survival rate was 50%. There was one episode of rebleeding 60 days after stent removal. 

Dechêne et al. recently reported their experience using SEMS (SX-Ella Danis; stent Ella-CS, Hradec Kralove, Czech Republic) in 8 patients with refractory esophageal variceal bleeding events [25]. One patient was treated twice over a period of 7 months. Source of bleeding was confirmed to be esophageal varices in all cases and excluded patients bleeding from gastric varices. Balloon tamponade was used in 3 patients prior to transportation to the tertiary center. SEMSs were placed successfully in all patients. The proper stent position and cessation of bleeding was confirmed by upper endoscopy. Control of bleeding was successful in 8/9 bleeding episodes (one patient died within five days of presentation). All stents were removed successfully after a median of 11 (7–14) days with no immediate rebleeding. No stent migration occurred in this series. The only complication related to the stent was compression of the left main bronchus which was treated successfully with stent removal. Definitive therapy was feasible in two patients (OLT and TIPS) and no rebleeding was noted in this group. TIPSs were contraindicated in the remaining six patients due to hepatic failure, hepatic encephalopathy, or hepatocellular carcinoma. Three patients had rebleeding 1, 2, and 9 days after stent removal. The 60-day survival rate was 25%.

Standard fully covered SEMSs have also been used successfully for the management of esophageal tears caused by Sengstaken-Blakemore tube or banding. These applications further confirm the important role of SEMS in the successful management of iatrogenic esophageal injuries [21, 26, 27].

## 4. Indications and Benefits

Limited data suggests that specially designed SEMS (SX-Ella Danis stent) can effectively stop refractory bleeding from esophageal varices (Table 1). This stent is usually deployed over an endoscopically placed wire without the need for radiological control. Limited data suggest that stent can also be delivered even without endoscopic assistance and without the need for continued endotracheal intubation compared to BT. Oral intake and nutrition are maintained. Stents can be left in place for as long as two weeks, allowing for improvement in liver function and institution of secondary prophylaxis before removal. Overall, compared to BT, SEMSs appear to be as effective, easier to insert and are associated with a lower risk for complications. Repeat endoscopy, if needed, can be performed while stent is in place.

## 5. Limitations and Complications

Stent placement requires appropriate training and expertise. Gastric varices will not be adequately compressed by the stent and persistent variceal bleeding after stent placement should raise the suspicion for presence of bleeding gastric varices. Appropriate precautions to prevent aspiration are needed since the stent is positioned at the gastroesophageal junction. Distal stent migration into the stomach was observed frequently but was not associated with apparent complications. Stent-related compression of the left main bronchus was reported in one patient and was treated successfully with stent removal [25, 28]. No reports of esophageal wall hyperplasia as seen with other esophageal indications have been published. However, all stents need to be removed within 1-2 weeks to minimize the risk of migration and wall injury or reaction.  Table 2 summarizes indications, efficacy, benefits, and limitations of the current rescue therapies in refractory esophageal variceal bleeding.

## 6. Conclusion

Current rescue therapies for bleeding esophageal varices are effective in stopping the bleeding in the majority of patients (Table 2). In some patients, standard therapies may fail, are associated with serious complications, or may not be possible to use because of patient characteristics. SEMS placement using especially designed stent (SX-Ella Danis stent, currently not available in USA) is a new promising alternative therapeutic technique that can be used in patients with refractory esophageal variceal bleeding. Patients who failed initial standard therapy, have contraindications, or are unsuitable for those therapies are good candidates at this time. Limited data suggests that when stent and expertise are available, these SEMSs can be considered as an alternative to BT. Use of SEMS is considered only as a bridge allowing stabilization of the patients until more definitive therapy is performed (banding, TIPS, shunt surgery, or liver transplant). The applicability of data using the specialized SEMS (SX-Ella Danis stent) to other currently available fully covered esophageal stents is not clear at this time. However, all fully covered SEMS can be used to manage iatrogenic esophageal injuries associated with the use of BT or banding. Further studies are needed to confirm safety and efficacy of SEMS in a large group of patients with bleeding esophageal varices and to establish their role in the management of such patients. 

## Figures and Tables

**Table 1:** Published series using SEMS for refractory esophageal variceal bleeding.

	Year of publication	Number of patients	% of success of placement of SEMS	% of control of bleeding	Stent migration	Recurrent bleeding	Local complications	Mortality
Hubmann et al. [23]	2006	20	100%	100%	25%	0	One minor esophageal ulcer.	Two died within 5 days.
Zehetner et al. [24]	2008	39	100%	100%	18%	0	One minor esophageal ulcer.	30-day mortality 26.5%.
Wright et al. [21]	2010	10	90%	70%	Not reported.	1 rebleeding at 60 days.	Small proximal esophageal ulcer.	42-day mortality 50%.
Dechêne et al. [25]	2012	8	100%	88%	0%	3 rebleeding.	Compression of left main bronchus.	60-day mortality 75%.

**Table 2:** Rescue therapies for refractory esophageal variceal bleeding.

Modality	Candidate	Efficacy in controlling bleeding	Complications	Limitation
BT	Refractory esophageal bleeding as bridge to definitive therapy.	More than 80% but tube should be removed within 24 hours.	Potentially lethal complications including esophageal perforation aspiration and pneumonia.	Limited efficacy and high complication rate in in-experienced hands. Temporary measure

Surgery	Acute variceal bleeding unresponsive to medical and endoscopic therapy.	Heterogeneous group but generally very effective.	Hepatic encephalopathy. Liver decompensation.	Requires expertise with exception of modified Sugiura procedure.

TIPS	Acute variceal bleeding unresponsive to medical and endoscopic therapy.	More than 90%.	Hepatic encephalopathy.	Limited availability
			Liver decompensation.	Occlusion and stenosis.
				Not suitable or contraindicated in many patients.

SEMSs	Refractory esophageal bleeding as bridge to definitive therapy.	70–100% and stent can be left in place for as long as 2 weeks.	Minor esophageal ulcer.	Temporary measures
			Migration.	Require a repeat endoscopy for removal.
			Compression of left main bronchus.	

## Abbreviations

BT: Balloon tamponade
TIPS: Transjugular intrahepatic portosystemic shunt
SEMS: Self-expandable metal stent.
